# Disaggregation of Islet Amyloid Polypeptide Fibrils as a Potential Anti-Fibrillation Mechanism of Tetrapeptide TNGQ

**DOI:** 10.3390/ijms23041972

**Published:** 2022-02-10

**Authors:** Raliat O. Abioye, Ogadimma D. Okagu, Chibuike C. Udenigwe

**Affiliations:** 1 Department of Chemistry and Biomolecular Sciences, Faculty of Science, University of Ottawa, Ottawa, ON K1N 6N5, Canada; rabio069@uottawa.ca (R.O.A.); ookag095@uottawa.ca (O.D.O.); 2 School of Nutrition Sciences, Faculty of Health Sciences, University of Ottawa, Ottawa, ON K1H 8M5, Canada

**Keywords:** islet amyloid polypeptide, aggregation, disaggregation, fibril formation, bioactive peptides, biomolecular interaction, antidiabetic agents, nutraceuticals

## Abstract

Islet amyloid polypeptide (IAPP) fibrillation has been commonly associated with the exacerbation of type 2 diabetes prognosis. Consequently, inhibition of IAPP fibrillation to minimize β-cell cytotoxicity is an important approach towards β-cell preservation and type 2 diabetes management. In this study, we identified three tetrapeptides, TNGQ, MANT, and YMSV, that inhibited IAPP fibrillation. Using thioflavin T (ThT) fluorescence assay, circular dichroism (CD) spectroscopy, dynamic light scattering (DLS), and molecular docking, we evaluated the potential anti-fibrillation mechanism of the tetrapeptides. ThT fluorescence kinetics and microscopy as well as transmission electron microscopy showed that TNGQ was the most effective inhibitor based on the absence of normal IAPP fibrillar morphology. CD spectroscopy showed that TNGQ maintained the α-helical conformation of monomeric IAPP, while DLS confirmed the presence of varying fibrillation species. Molecular docking showed that TNGQ and MANT interact with monomeric IAPP mainly by hydrogen bonding and electrostatic interaction, with TNGQ binding at IAPP surface compared to YMSV, which had the highest docking score, but interact mainly through hydrophobic interaction in IAPP core. The highly polar TNGQ was the most active and appeared to inhibit IAPP fibrillation by disaggregation of preformed IAPP fibrils. These findings indicate the potential of TNGQ in the development of peptide-based anti-fibrillation and antidiabetic nutraceuticals.

## 1. Introduction

Islet amyloid polypeptide (IAPP), or amylin, is a highly amyloidogenic, 37-residue polypeptide that has been strongly linked to the exacerbation of type 2 diabetes (T2D) prognosis [1,2]. Mature IAPP fibrils induces β-cell cytotoxicity due to their permeation through the cell membrane, resulting in intracellular ion imbalance, formation of reactive oxidative species, membrane disruption, and other deleterious effects [1,3]. Co-packaged and co-secreted along with insulin from pancreatic β-cells, IAPP plays many roles in regulating glucose metabolism [1,4,5,6]. As a result, disease treatments that target the upregulation of insulin secretion may inadvertently encourage IAPP fibrillation due to the increased production and secretion of IAPP, resulting in the additional loss of β-cell mass due to IAPP fibril-induced cytotoxicity [7].

The mechanism governing IAPP fibrillation-induced β-cell toxicity is not well understood, and many hypotheses point to varying species as the source of cytotoxicity [1,8]. The fibril-induced toxicity hypothesis proposes β-cell cytotoxicity due to mechanical stress imparted on cell membranes by the insoluble IAPP mature fibrils [8]. This results in membrane disruption, causing imbalance in water and ion hemostasis, triggering apoptosis and cell death [1,5,8]. In fact, IAPP mature fibrils, in the absence of oligomeric species, caused notable β-cell cytotoxicity [9,10]. Alternatively, soluble oligomeric species could also cause membrane disruption through the formation of pores, leading to a dysregulated flow of ions and membrane disruption, thus triggering apoptotic pathways [11]. In this hypothesis, insoluble fibrils are regarded as relatively inert and incapable of exerting cytotoxic effects on β-cells. Recently, it has been highlighted that reduced proteolytic turnover of IAPP as a result of impaired proteasome and autophagy machinery could also facilitate the accumulation of toxic hIAPP species and encourage β-cell death [12]. As such, stabilization of monomeric IAPP is the most effective approach for inhibiting IAPP fibrillation. This approach discourages self-association of IAPP, thus preventing fibrillation and oligomeric or fibrillar-induced cytotoxicity, while also retaining the intrinsic function of IAPP. Some natural compounds, such as polyphenols, alkaloids, and peptides, have demonstrated promising anti-fibrillation activities [13,14]. This property may be a contributing mechanism of the antidiabetic properties of some of the compounds. Therefore, IAPP fibrillation provides an important target for the development of new antidiabetic nutraceutical compounds.

Bioactive peptides are promising candidates for use as inhibitors of IAPP fibrillation. In fact, many food-derived peptides have demonstrated physiological antidiabetic properties [15,16], but their bioactivity mechanisms have yet to be linked to IAPP fibrillation. Furthermore, peptides are more easily modifiable to enhance their biostability, bioavailability, functionality, and bioactivity. To date, only a few unmodified linear peptides, such as pentapeptide FLPNF, have been reported as inhibitors of IAPP fibrillation [1]. There is equally a dearth of information regarding the important structural requirements of peptides for increasing inhibitor potency. This underscores the need for library screening and rational design for the identification of natural peptide inhibitors of IAPP fibrillation. Therefore, the objective of this study was to evaluate the effect and mechanism of three tetrapeptides as inhibitors of IAPP fibrillation. The findings provide important insight into a new structural motif for an effective inhibitor that can disaggregate IAPP fibrils, and platform for the discovery of peptide-based antidiabetic nutraceuticals.

## 2. Results

### 2.1. Thioflavin T Fluorescence Kinetics

**Figure 1:**
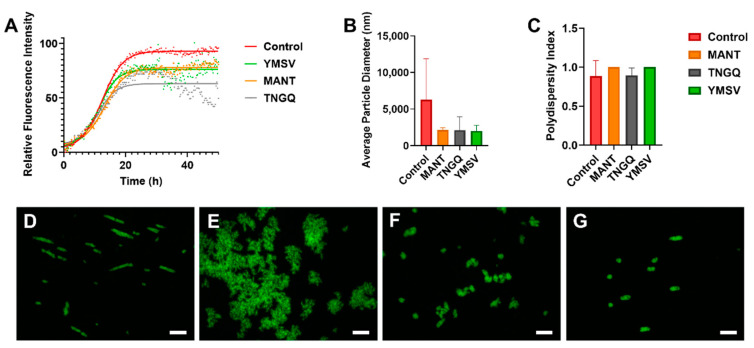
(**A**) Thioflavin-T fluorescence kinetics of IAPP fibrillation in the absence (control) and presence of tetrapeptides MANT, TNGQ, and YMSV. (**B**) Average particle size diameter (nm) and (**C**) polydispersity index of IAPP in the absence (control) and presence of MANT, TNGQ, and YMSV in the late stationary phase of fibrillation. ThT fluorescence microscopy of IAPP in the (**D**) absence (control), and presence of peptides (**E**) MANT, (**F**) TNGQ, and (**G**) YMSV. Scale bars represent 50 μm.

Several random linear peptides from our library were screened for their anti-IAPP fibrillation activity. Thereafter, based on ThT fluorescence kinetic parameters, tetrapeptides TNGQ, YMSV, and MANT were selected for subsequent analyses. ThT is a dye that strongly fluoresces upon binding to β-sheet rich regions within peptide aggregates. Thus, the stronger the fluorescence intensity, the higher the amount of β-sheets present. Based on ThT fluorescence kinetics, the three tetrapeptides alone did not form fibrils (data not shown), but their presence decreased the maximum fluorescence intensity (F_max_) of IAPP. Notably, TNGQ showed the highest inhibitory activity in reducing ThT fluorescence, with 32% lower F_max_ compared to the IAPP control at the end of IAPP fibrillation (Figure 1A, Table 1). In terms of physicochemical properties, TNGQ also had the lowest instability, aliphatic, and hydrophobicity indices, as well as the highest Boman index compared to MANT and YMSV (Table 1).

Furthermore, TNGQ and YMSV had more pronounced effects in reducing the elongation phase of fibrillation, by having the largest elongation constant (Table 1). Furthermore, TNGQ caused a gradual decrease in fluorescence intensity from 35–49 h of incubation, with the largest decrease of 53.8% at 48.75 h relative to control (Figure 1A). In comparison, much lesser decreases in fluorescence intensities (14.1% and 8%) were observed for YMSV and MANT, respectively, at the same time point (Figure 1A). YMSV and MANT also reduced the F_max_, but to a lesser extent (16% and 18%, respectively) than TNGQ compared to control F_max_ (Table 1). MANT slightly increased the *lag time* compared to the other tetrapeptides but did not have an apparent effect on the elongation constant compared to control (Table 1).

### 2.2. Fluorescence Morphology of IAPP Fibrils

Fluorescence imaging of ThT-stained IAPP samples was used for preliminary assessment of the morphology of IAPP fibrils in the late stationary phase. According to the images, TNGQ and YMSV resulted in the formation of smaller IAPP aggregates compared to IAPP control (Figure 1D,F,G). The ThT-fluorescent aggregates were particularly lesser in the presence of TNGQ. Conversely, MANT resulted in the formation of larger aggregates compared to IAPP control (Figure 1D,E).

### 2.3. IAPP Fibrillation Progression

Average particle size and polydispersity index were determined by dynamic light scattering (DLS) to further assess fibrillation. MANT, TNGQ, and YMSV alone had particle size diameters of 409.8, 380.8, and 223.3 nm, respectively compared to the 6279 nm average diameter of IAPP control. At the end of fibrillation, the peptides reduced the average particle size of IAPP by 66.7% compared to the control (Figure 1B), although the change was not statistically significant (*p* > 0.05). The polydispersity index was high (>0.7), with no significant difference between the peptide-treated and control IAPP samples at 48 h of incubation (Figure 1C).

### 2.4. IAPP Secondary Structure during Fibrillation

**Figure 2:**
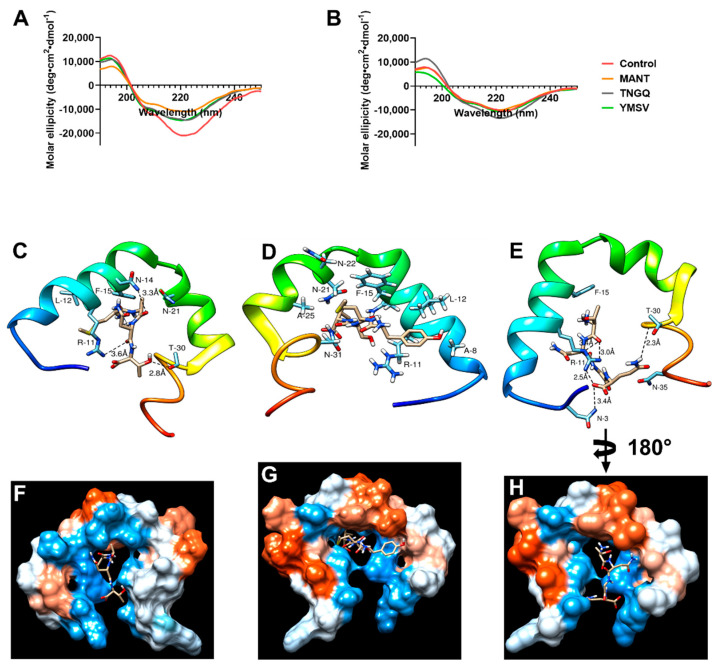
Circular dichroism spectra of IAPP in the absence (control) and presence of MANT, TNGQ, and YMSV at (**A**) the initial time point (0 h) and (**B**) late stationary phase (48 h) of fibrillation. Docking scheme showing intermolecular interactions between (**C**) MANT, (**D**) YSMV, or (**E**) TNGQ and monomeric IAPP at the various hydrophobic and hydrophilic regions (**F**–**H**), respectively. Kyte–Doolittle scale was used to evaluate hydrophobicity with colors ranging from dodger blue (the most hydrophilic) to white 0.0 to orange-red (the most hydrophobic).

Circular dichroism was used to evaluate the effect of the tetrapeptides on IAPP secondary structure and the potential mechanism of inhibition. As expected, a strong negative peak at 220 nm with a shoulder at approximately 206 nm was observed in IAPP control at the initial time point due to α-helical structure of monomeric IAPP (Figure 2A). This secondary structure content was decreased by binding the peptides at time 0 h. As revealed by molecular docking, TNGQ interacts with monomeric IAPP mainly through its hydrophilic and charged amino acid residues. Interactions with N-3, R-11, N-35, and T-30 were facilitated via five hydrogen bonding and electrostatic interactions at the hydrophilic region in the surface of IAPP (Figure 2E,H). This interaction yields a relative docking score of −109.1. MANT, with a docking score of −107.7, interacts with relatively charged and hydrophilic residues at the protein core–surface interface and undergoes three hydrogen bonding with R-11, N-14, and T-30 (Figure 2C,F). On the other hand, YMSV (Figure 2D,G), being the most hydrophobic tetrapeptide, undergoes mainly hydrophobic interactions with IAPP and lies completely in the core of IAPP, where it interacts with hydrophobic residues such as A-8, L-12, F-15, and A-25 while maintaining electrostatic contacts with N-21, N-22, and N-31. This results in a docking score of −128.4. These relative binding affinities are calculated with a high accuracy molecular docking program, HPEPDOCK web server, based on SIMPLEX minimization algorithm binding scores and specifically designed for studying the nature and strength of protein-peptide and protein-protein interactions. In HPEPDOCK docking score, the original scoring function is replaced with an iterative knowledge-based scoring function for protein-peptide and protein-protein interactions. The binding affinities are based on the contributions of intermolecular forces, such as electrostatic, hydrophobic, Van der Waals, hydrogen bonding, entropy, and conformational state of the ligand [17,18]. The higher their negative values, the greater the strength of the interaction, the stability of the complex formed, and the potential to trigger physiological response at low concentrations. At 48 h, following fibrillation, the relative α-helical content of IAPP control decreased by 14.7% with concomitant increase in the undefined secondary structure content by 14.2% (Figure 2B).

### 2.5. IAPP Fibril Morphology

**Figure 3:**
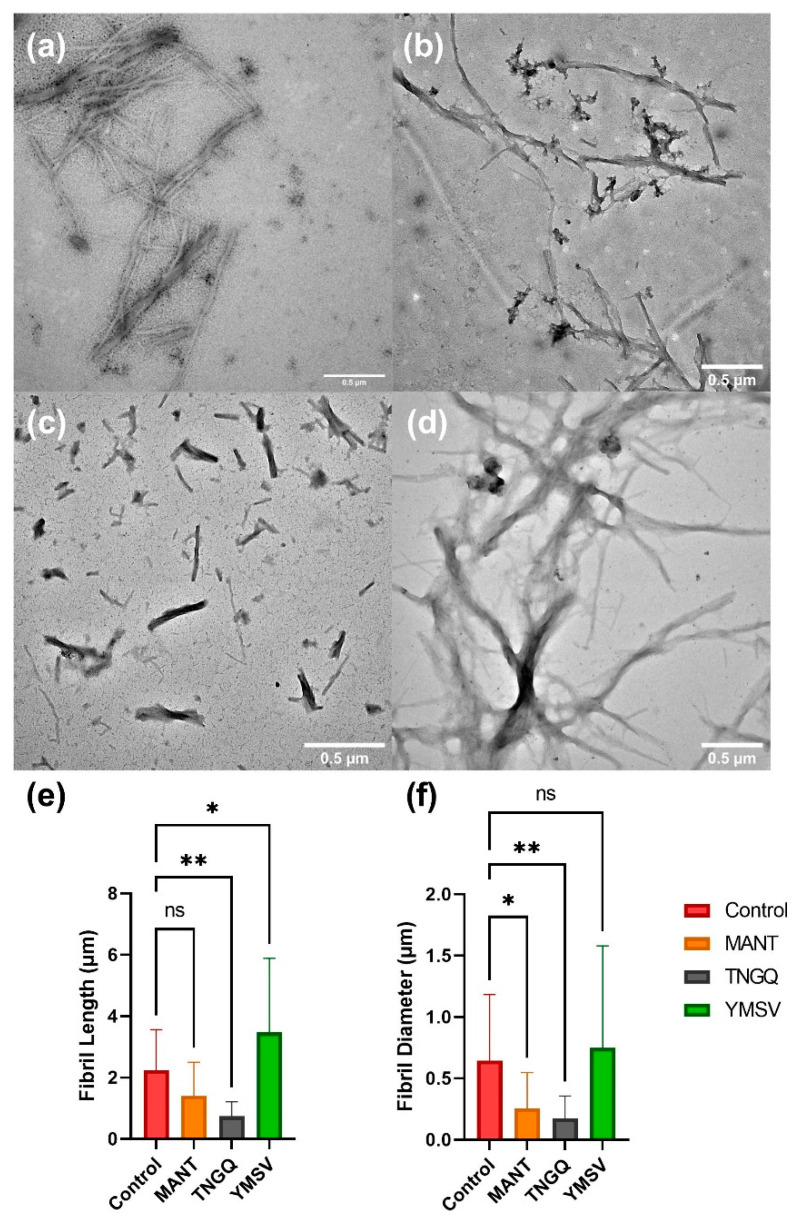
Transmission electron microscopy images of (**a**) IAPP control, and IAPP in the presence of (**b**) MANT, (**c**) TNGQ, and (**d**) YMSV, after 48 h incubation. IAPP fibril (**e**) length and (**f**) diameter quantified using ImageJ software (*n* = 24); ns = not significant (*p* ≥ 0.05), * = significant (0.01 > *p* > 0.05), and ** = very significant (0.01 > *p* > 0.001). Scale bars represent 0.5 μm.

To confirm the observed effects of the tetrapeptides on IAPP fibrillation, TEM was employed to evaluate the fibrillar morphology. The tetrapeptides showed different effects on IAPP fibril morphology. MANT significantly reduced the fibril diameter and density, but not the fibril length, compared to the control (Figure 3a,b). On the other hand, TNGQ significantly reduced both fibril length and diameter (Figure 3c), thus confirming its effect as the most potent inhibitor evaluated. Lastly, YMSV significantly increased the fibril length, and not the diameter, resulting in distinctly different fibril morphology compared to IAPP control (Figure 3d).

### 2.6. Disaggregation of Pre-Formed IAPP Fibrils

**Figure 4:**
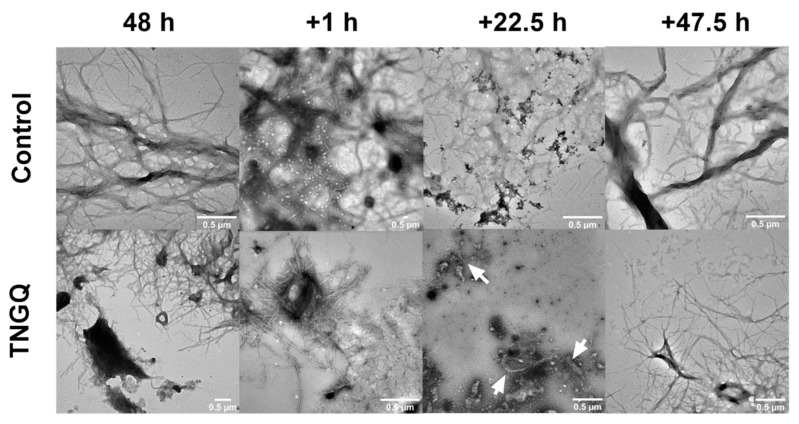
Transmission electron microscopy images of pre-formed IAPP fibrils in the absence (control) and presence of TNGQ at 48 h and additional 1, 22.5, and 47.5 h post-incubation. Scale bars represent 0.5 μm. Arrows indicate presence of fibrils amongst amorphous aggregates.

To observe the potential disaggregation effect of TNGQ, pre-formed IAPP fibrils were incubated with TNGQ and imaged periodically. As expected, extensive fibrillar networks were observed after 48 h of incubation in both the control and TNGQ-treated IAPP fibrils (Figure 4). An hour after the addition of TNGQ, amorphous aggregates were observed on the outer perimeter of the fibrils, which was absent in the control fibrils (Figure 4). At 22.5 h, TNGQ, further reduced the fibrillar networks to form more amorphous aggregates intercalated with fibrils (arrows) (Figure 4). After 47.5 h post-addition of TNGQ, mature fibrils are observed, albeit less extensive than the control, which had denser and larger fibrillar networks (Figure 4).

### 2.7. In Silico Drug-Likeness of the Peptides

In silico ADME/Tox analysis was performed to evaluate the pharmacokinetic and safety properties of the tetrapeptides. All three peptides were predicted to be non-toxic and lack the ability to inhibit cytochrome P450 3A4 (Table 2). Moreover, the peptides were identified as P-glycoprotein substrates. However, all the peptides were predicted to have low gastrointestinal absorption. High molecular flexibility indicated by the rotatable bond (ROTB) count greater than 10 and topological polar surface area (TPSA) greater than 140 Å^2^ indicate low oral bioavailability of the peptides (Table 2) [19,20,21]. Additionally, the tetrapeptides each had over 12 total hydrogen bond acceptors (HBA) and hydrogen bond donors (HBA), which also indicates potential low oral bioavailability [21].

## 3. Discussion

Based on IAPP fibrillation inhibitor screening studies of a library of randomly selected peptides, three active tetrapeptides were selected to further investigate their effectiveness and mechanisms of inhibition. TNGQ exhibited the strongest inhibitory effects, primarily influencing the stationary phase of IAPP fibrillation (Figure 1A, Table 1). The Boman index (Table 1) indicated that only TNGQ had high protein binding potential, and yet it had a lower IAPP docking score compared to YMSV. Notably, only TNGQ bound outside of the hydrophobic pocket in the amyloidogenic region (Figure 2E,H). In fact, TNGQ binding favored interactions with N3, R11, and F15 of the membrane-binding domain, and T30 and N35 of the self-association/C-terminus region of IAPP. This binding pattern of TNGQ reflected in the relative secondary structures present. After 48 h of incubation, TNGQ maintained the relative α-helical content of IAPP compared to the control, MANT, and YMSV, which showed marked decreases relative to the initial timepoint (Figure 2A,B). It is possible that TNGQ binding with monomeric IAPP stabilized the α-helical conformation to alter the thermodynamic equilibrium between IAPP species against fibril formation. The complexation of TNGQ and monomeric IAPP reduces the relative amount of unbound monomeric IAPP, thus halting fibrillation progression and delaying the formation of extensive IAPP fibrillar networks normally found in the stationary phase (Figure 3a,c). Similarly, amphipathic heptapeptide KPWWPRR-NH_2_ was reported to arrest IAPP fibril elongation [25]. The hydrophobic regions of the heptapeptide bound the non-polar regions of IAPP while the hydrophilic regions disrupted elongation by interfering with the formation of H-bonds required for association of monomeric IAPP on the fibril growth end [25]. Consequently, significantly shortened fibrils with smaller diameters were observed in our study (Figure 3c,e–f). This confirms the strong anti-fibrillation propensity of TNGQ and demonstrates the importance of physicochemical properties of peptides on inhibition mechanism and potency.

The formation of the IAPP-TNGQ complex provides additional insight on the mechanism governing the disaggregation of preformed fibrils (Figure 4). IAPP fibrils were disaggregated starting from the outer regions of the extensive fibrillar network as soon as 1 h after the addition of TNGQ (Figure 4). The disaggregation effects intensified at 22.5 h of TNGQ treatment where amorphous aggregates are observed to surround the fibrils (Figure 4). These aggregates are similar in shape and size to that observed in the initial timepoint of IAPP control (data not shown). This suggests the formation of monomeric or oligomeric species following fibrillar disaggregation by TNGQ. Similar structures have been identified as oligomeric species of α-synuclein [26]. In the process of nucleation, self-assembly is driven by aromatic interactions within the central part of IAPP, with long-lasting interactions between 14-NFLVH-18 and 25-AILSST-30 driving the formation of β-sheets [27]. Additionally, the prenuclear β-hairpin conformation is an important structural conformation that promotes dimerization, primarily driven by the C-terminal region residues 17-VHSSNNFGAIL-27 and 29-STNVGSTN-35 [28]. This provides a mechanistic insight into the IAPP fibril disaggregation effects of TNGQ. As fibrillation is a thermodynamically driven process, species equilibrium will strongly influence fibrillation progression [29]. Formation of the IAPP-TNGQ complex may have shifted the mature fibril/monomeric IAPP equilibrium towards the formation of monomeric IAPP. Visually, this manifested as the disaggregation of preformed fibrils (Figure 4) wherein monomeric IAPP liberated from the growing ends of the fibrils are used to re-establish equilibrium. This can also explain why the addition of TNGQ inhibited fibrillation even though it did not elongate the lag phase. This finding provides a unique perspective to fibrillation inhibitor design, which hitherto has focused largely on association with the amyloidogenic region [30,31,32,33]. Recent investigations on the development of peptide inhibitors have started considering non-amyloidogenic regions, namely the C-terminal self-association region, and N-terminal membrane-binding domain due to their important roles in fibril formation and toxicity [34,35]. Consequently, the ability of TNGQ to bind within the N-terminal membrane-binding domain may reduce the cytotoxic effects commonly observed with fibrillation by preventing membrane association and subsequent pore formation. For instance, pentapeptide FLPNF, which binds within the membrane-binding domain and self-association region of the N- and C-terminus, respectively, was reported to increase the viability of cultured rat insulinoma cells [35] This illustrates the potential of targeting the non-amyloidogenic regions of IAPP as an alternative mechanism in the development of novel anti-fibrillation agents.

Amino acid composition of peptides can affect the inhibitor potency and mechanism of IAPP fibrillation inhibition. Previous studies have reported that hydrophobic and aromatic interactions are the major forces driving IAPP fibrillation [36,37]. Additionally, some phenolic compounds were hypothesized to inhibit fibrillation via π-π and hydrophobic interactions within the amyloidogenic core of IAPP [1,13,37]. Thus, we expected that hydrophobicity would correlate with inhibitor potency. However, YMSV was the most hydrophobic of the tetrapeptides tested, and only moderately inhibited fibrillation (Figure 1A, Table 1). Incidentally, YMSV also has the lowest protein binding potential of the three tetrapeptides (Table 1), despite having the highest IAPP docking score. This suggests a wider range of structural requirements for potent inhibitors, beyond hydrophobicity and aromatic content, and that favorable inhibitor binding to IAPP does not guarantee increased activity. Future studies on the effect of peptide sequence and physicochemical properties on inhibition of IAPP fibrillation, for example through the development of scrambled peptide sequences, could be done to further investigate this relationship. MANT, which had the least inhibitor strength, slightly inhibited the late stationary phase of IAPP fibrillation due to the lower F_max_ compared to IAPP control (Figure 1A, Table 1). However, ThT fluorescence imaging in the late stationary phase revealed that MANT led to the formation of larger aggregates compared to the control (Figure 1D,E). This may be due to the limitations of ThT fluorescence imaging wherein non-fibrillar species may result in fluorescence. Nonetheless, TEM imaging indicated the formation of fibrillary networks with significantly smaller diameter but similar in length compared to IAPP control (Figure 3f). This confirms the ThT fluorescence kinetics results, which showed that MANT was not a strong inhibitor of fibrillation compared to TNGQ (Figure 1A, Table 1).

Despite the variable inhibitory activities observed with the three tetrapeptides, IAPP fibrillation still occurred in each sample, indicated by the increase in particle size beyond a radius of 1100 nm, which has been reported to be the average size of IAPP fibrils at the end of late stationary phase [38]. Although the IAPP particle size did not differ significantly, IAPP in the presence of MANT, TNGQ, and YMSV reduced the average diameter compared to the control (Figure 1B). The exceedingly high particle size of uninhibited IAPP suggests the formation of extensive fibrillar networks, which were suppressed in treated samples due to the variable inhibitory activities of the tetrapeptides. Furthermore, the high polydispersity index indicated high heterogeneity of species present within the samples, which is characteristic for IAPP fibrillation (Figure 1C). The heterogeneity could also be a result of fibril disaggregation resulting in smaller species, or an artifact of active fibrillation inhibition through the formation of IAPP-peptide complexes [38].

In addition to being the strongest inhibitor, TNGQ also showed promising biostability and pharmacokinetic properties. It was predicted to be more biostable in the gastrointestinal tract than the others, as it lacks the recognition and cleavage sites for pepsin, trypsin, and chymotrypsin. The ADME profile also indicated that the non-toxic tetrapeptide can be rapidly metabolized and removed from cells in the body, thus suggesting its suitability for human consumption. However, TNGQ and the other peptides were predicted to have low oral bioavailability based on their ROTB, HBA, and TPSA [20,21]. This factor must be addressed prior to conducting *in vivo* studies in order to achieve significant IAPP fibrillation inhibitory effects in the pancreas. TNGQ can be found naturally, e.g., in rice oryzain beta chain (f177-180; accession no. P25777 (ORYB_ORYSJ)); thus, future research should include the development of sustainable processing methods, e.g., enzymatic hydrolysis or fermentation, to release the bioactive peptide from its parent proteins.

## 4. Materials and Methods

### 4.1. Materials

Human IAPP (amylin (1–37), human, >95% pure) modified with an amidated C-terminus and Cys2-Cys7 disulfide bond was purchased from AnaSpec (Fremont, CA, USA). 1,1,1,3,3,3-Hexafluoro-2-propanol (HFIP), thioflavin T (ThT), Tris base, sodium phosphate monobasic, and sodium phosphate dibasic were purchased from MilliporeSigma (Oakville, ON, Canada). Tetrapeptides MANT (98.7% purity), YMSV (95.5% purity), and TNGQ (95.7% purity) were synthesized by GenScript (Piscataway, NJ, USA). UranyLess counterstain was purchased from Electron Microscopy Sciences (Hatfield, PA, USA).

### 4.2. IAPP Preparation

For inhibition experiments, 1 mg IAPP was dissolved in 10 mL HFIP on ice for 10 min and then separated into 1 mL aliquots with a final concentration of 0.1 mg/mL per tube. Samples were sonicated in an ice bath for 30 min and centrifuged at 14,000× *g* for 30 min to disaggregate preformed fibrils. Each tube was frozen overnight at −80 °C and lyophilized the following day. Peptide films were stored at −80 °C until use. The films were reconstituted with 12.5% (*v/v*) HFIP in 1 M Tris buffer (pH 7.4) immediately prior to experimentation. For circular dichroism analysis, 100 mM phosphate buffer (pH 7.4) was used as the diluent instead.

### 4.3. Thioflavin T (ThT) Fluorescence Assay

To observe fibrillation kinetics, Thioflavin T (ThT) fluorescence assays was performed in triplicate. In black 96-well microplates with clear bottoms, 5 µM IAPP was mixed with 5 µM tetrapeptide and 10 µM ThT in 1 M Tris buffer (pH 7.4). Plates were sealed with Parafilm to minimize evaporation and fibrillation was monitored via fluorescence kinetic measurements. Fluorescence intensity was measured at λ_ex_ = 430 nm and λ_em_ = 480 nm using the Spark multimode microplate reader (Tecan, Stockholm, Sweden). Under the kinetic mode, bottom measurements were taken every 15 min for 49 h at 37 °C. Results were presented as means and kinetic parameters and were calculated using the non-linear regression Boltzmann sigmoidal function from GraphPad Prism version 9.2.0 for Windows (GraphPad Software, La Jolla, CA, USA) using the equation:

(1) $$F_{obs}=\frac{F_{i}+F_{f}}{1+exp\;\left(\frac{t_{50}-t_{obs}}{\tau }\right)\;}$$

*F_obs_* is the log fluorescence intensity observed at time *t_obs_*, *F_i_* and *F_f_* are the initial and final ThT fluorescence, respectively, *t*_50_ is the time taken to reach half the elongation phase, and *τ* is reciprocal of the kinetic elongation constant, K, an apparent rate constant that describes the growth of fibrils during the elongation phase. Additionally, the *lag time* (h) of IAPP fibrillation was calculated using the equation:

(2) $$Lag\;time=t_{50}-2\tau$$

### 4.4. Circular Dichroism (CD) Spectroscopy

The secondary structure of IAPP was analyzed using the Jasco J-715 Circular Dichroism spectrophotometer (Jasco Corp., Tokyo, Japan). Each sample consisted of 20 µM IAPP and 20 µM tetrapeptide in 10 mM phosphate buffer (pH 7.4). Two time points, initial (0 h) and final (48 h), were measured using a quartz cuvette with a path length of 1 mm at room temperature in nitrogen gas. Samples were incubated at 37 °C in between the time point measurements. Three scans were recorded and averaged at a wavelength range of 200–250 nm at a scanning speed of 100 nm/sec. Baseline subtraction was done using a phosphate buffer blank and results were converted to mean residue ellipticity (deg × cm^2^ × dmol^−1^) using a mean residue weight of 105.49 Da and IAPP concentration of 0.078 mg/mL. All data processing was performed using CDToolX [39]. Plotting and smoothing of resulting data was achieved with GraphPad Prism version 9.2.0 for Windows (GraphPad Software, La Jolla, CA, USA). Calculation of the secondary structure contents was performed using the CD fitting software, BeStSel [40].

### 4.5. Molecular Docking of IAPP-Tetrapeptide Interaction for Determination of Substantive Binding Site and Relative Binding Affinity

Molecular docking was performed by uploading the crystal structure of IAPP (PDB code: 2L86) retrieved from RCBS protein data bank and the amino acid sequence of the tetrapeptides to HPEPDOCK web serve. MODPEP program was used for conformational refinement of the peptides [17]. Chimera UCSF software version 1.15 [41] equipped with Autodock Vina package version 1.1.2 [42] was used for preparation and structural optimization of the crystal structure of IAPP prior to docking. This involves eliminating solvents, adding Gastejger charges and polar hydrogen, ignoring non-standard amino acid residues, and energy minimization to reduce internal clashes. Docking results of the top model were analyzed and visualized with Chimera for intermolecular interactions within 3 Å.

### 4.6. Fluorescence Microscopy

An initial sample consisting of IAPP and tetrapeptide at a ratio of 1:1, with 5 µM of each component, was added to 1 M Tris buffer (pH 7.4) and incubated at 37 °C for 48 h. Thereafter, each sample was stained with 10 µM ThT and kept in the dark for 2 min at room temperature. The samples were then mounted on microscope slides and secured with a coverslip prior to imaging. The Axio Imager 2 fluorescence microscope equipped with an Axiocam 506 camera (Carl Zeiss, Germany) was used to image the samples, using the fluorescein isothiocyanate channel with λ_ex_ = 495 nm and λ_em_ = 519 nm. Images were subsequently processed using the Zen 2.3 pro software (Carl Zeiss, Germany). Aggregate length and diameter measurements were done using the ImageJ software (NIH, Bethesda, MD, USA) [43].

### 4.7. Dynamic Light Scattering (DLS)

The average particle size and polydispersity index of uninhibited IAPP and IAPP incubated with the tetrapeptides were determined using the Nano-ZS Zetasizer (Malvern Instruments Ltd., Malvern, UK). Samples, consisting of IAPP:tetrapeptide ratio of 1:1 with 5 µM of each component in 1 M Tris buffer (pH 7.4), were incubated at 37 °C for 48 h. Particle size and polydispersity index measurements were subsequently taken in triplicate.

### 4.8. Transmission Electron Microscopy (TEM)

TEM was used to observe the effect of the peptides on IAPP fibril morphology. Samples, consisting of 5 µM IAPP and 5 µM tetrapeptide in 1 M Tris buffer (pH 7.4), were incubated at 37 °C for 48 h. Thereafter, 10 µL of the mixture was placed on Parafilm and a 300-mesh Formvar-carbon-coated copper grid was placed on top of the droplet for 2 min. Excess sample was blotted with a Kimwipe. The grids were then counterstained with UranyLess for 1 min in the dark and dried. Grids loaded with sample were imaged using the JEM-1400Flash Electron Microscope (JEOL, Tokyo, Japan) at an accelerating voltage of 120 kV. Fibril length and diameter were measured using the ImageJ software (NIH, Bethesda, MD, USA) [43]. For fibril disaggregation analysis, TNGQ at a 1:1 ratio was added to pre-formed fibrils grown for 48 h at 37 °C. Sample aliquots were taken for TEM imaging at 1, 22.5, and 47.5 h after the addition of TNGQ to the preformed fibrils.

### 4.9. In Silico ADME/Tox and Physicochemical Properties of the Peptides

Physicochemical properties of the tetrapeptides (molecular weight, hydrophobicity, net charge at pH 7, Boman index, instability index, and aliphatic index) were calculated using the Peptides package in R. Gastrointestinal proteolytic stability was predicted using ExPASy PeptideCutter. Drug-likeness and pharmacokinetics of the peptides were evaluated using SwissADME (http://www.swissadme.ch/index, date accessed; 6 August 2021), which predicts the ADME (absorption, distribution, metabolism, and excretion) properties based on Lipinski’s rule-of-five [44]. For this analysis, SMILES strings of the peptides were retrieved from BIOPEP-UWM. Lastly, potential toxicity of the peptides was predicted using ToxinPred; a threshold of 0.0 (automated) was applied [23].

### 4.10. Statistical Analysis

Experiments were performed in triplicate and statistical analysis was performed using one-way analysis of variance with GraphPad Prism version 9.2.0 for Windows (GraphPad Software, La Jolla, CA, USA). Significant difference between the mean values was defined at *p* < 0.05 using the Dunnett’s multiple comparison test.

## 5. Conclusions

This study resulted in the discovery of three tetrapeptides, MANT, TNGQ, and YMSV, that present variable inhibitory effects on IAPP fibrillation. Through biomolecular analysis, potential mechanisms of inhibition and the effect of physicochemical properties of the peptides on activity were proposed. The weak activities of YMSV and MANT demonstrate that strong inhibitor binding to monomeric IAPP does not always translate to potent anti-fibrillation effects. TNGQ, the most active tetrapeptide studied, strongly inhibited IAPP fibrillation wherein it possibly bound IAPP monomers, thus preventing their subsequent attachment to the growing end of the fibril. Furthermore, the disaggregation of pre-formed IAPP fibrils is proposed to be the major anti-fibrillation mechanism of TNGQ. This highlights the importance of the monomer–fibril equilibrium in facilitating anti-fibrillation. The interplay between the physicochemical properties of peptides and anti-fibrillation mechanism and potency, an area that is currently understudied, provides an insight into structure–function relationships of the inhibitors. Lastly, studies to evaluate the antidiabetic and cell protective effects of TNGQ present a promising avenue for future applications.

## Figures and Tables

**Table 1:** Physicochemical properties and fibrillation kinetic parameters derived from ThT fluorescence assay of IAPP fibrillation in the absence (control) and presence of tetrapeptides MANT, TNGQ, and YMSV.

	Physicochemical Properties						Fibrillation Parameters				
	MW (Da)	Hydrophobicity	Net Charge	Boman Index	Instability Index	Aliphatic Index	F_max_	*t*_50_ (h)	τ	K	*Lag Time* (h)
Control	n/a	n/a	n/a	n/a	n/a	n/a	92.89	12.45	3.33	0.30	5.79
MANT	435.50	−0.13	−0.002	1.26	17.13	25	77.98	13.13	3.41	0.29	6.31
TNGQ	418.40	−2.03	−0.002	3.45	−67.65	0	63.11	11.00	2.89	0.35	5.22
YMSV	498.59	1.00	−0.003	−0.71	227.2	72.5	76.15	11.30	2.95	0.34	5.39

Abbreviations: MW, molecular weight; F_max_, maximum fluorescence intensity reached; *t*_50_, time taken to reach half elongation phase in hours; K, elongation constant; and n/a, not applicable. Boman index estimates peptide–protein interaction based on solubility properties of amino acid side chains. Instability index estimates stability of protein in a test tube (value less than 40 means that the protein is stable). Aliphatic index estimates thermostability of globular proteins based on the relative volume occupied by their aliphatic side chains.

**Table 2:** Absorption, distribution, metabolism, excretion, and toxicity (ADME/Tox) profile for tetrapeptides MANT, TNGQ, and YMSV to predict drug-likeness and suitability for human consumption.

Peptide	Physicochemical Properties				Toxicity	Lipophilicity		Drug-Likeness		Pharmacokinetics		
	ROTB (*n*) < 10	HBA (*n*) < 10	HBD (*n*) < 5	ESOL Log S	SVM Score (<0.0)	TPSA (Å^2^) < 140	ClogP (o/w) < 5	Bioavailability Score	Lipinski Filter	GIA	P-Gly Substrate	CYP3A4 Inhibitor
MANT	16	8	7	1.83 (HS)	−0.81 Non-toxin	239.24	−2.33	0.17	No	Low	Yes	No
TNGQ	16	9	8	3.32 (HS)	−0.72 Non-toxin	257.03	−3.94	0.17	No	Low	Yes	No
YMSV	17	8	7	0.23 (HS)	−0.89 Non-toxin	216.38	−0.39	0.17	No	Low	Yes	No

Abbreviations: ROTB (*n*), rotatable bonds; HBA (*n*), hydrogen bond acceptors; HBD (*n*), hydrogen bond donors; EOSL, estimated solubility [22] with solubility classes in bracket (HS, highly soluble); Toxicity SVM score (BIOPEP and ToxinPred), support vector machine score [23]; TPSA (Å^2^), topological polar surface area; CLogP (o/w) logarithm of compound partition coefficient between n-octanol and water; Bioavailability score, probability of F > 10% in rat [24]; Lipinski filter (based on Lipinski rules of 5, all peptides showed 3 violations); GIA, gastrointestinal absorption; P-gly substrate, permeability-glycoprotein substrate SVM model (SwissADME); and CYP3A4, cytochrome P450 3A4.

## Data Availability

Data supporting the findings are available within the article.
